# Bibliometric and visual analysis of suicide in aged people over the past 20 years

**DOI:** 10.3389/fpsyt.2025.1469853

**Published:** 2025-01-24

**Authors:** Shao-Kui Kan, Ying-Li Zhang, Xue-Xia Bai, Bo Peng

**Affiliations:** Depressive Disorders Ward I, Shenzhen Kangning Hospital/Shenzhen Mental Health Center, Shenzhen, China

**Keywords:** aged, suicide, depression, bibliometric, VOSviewer, Citespace

## Abstract

**Background:**

The rapid aging of the global population presents pressing public health challenges, notably an increase in suicide rates among older adults. Despite this critical issue, there is a scarcity of comprehensive assessments regarding the existing literature on suicide within this demographic. This study seeks to employ bibliometric analysis and knowledge mapping to elucidate prevailing research trends and the overall status of this field.

**Methods:**

We performed a comprehensive computer-based search of the Web of Science Core Collection to identify relevant articles and reviews concerning suicide in the elderly. A bibliometric analysis was conducted, examining various aspects including countries or regions, institutions, authors, journals, keywords, and references. This analysis utilized tools such as CiteSpace, VOSViewer, Pajek, and Excel 365 to facilitate a thorough assessment.

**Results:**

The analysis covered 1,116 publications from 2005 to 2024. The annual number of publications showed a fluctuating upward trend, with notable decreases in 2007, 2012, 2015, 2017, and 2022, and increases in 2009, 2013, 2016, and 2019, peaking at 121 in 2021, with citations reaching 4,741 in 2022, but declining since then. The United States stands out as the most productive and influential country in the field, boasting the highest number of publications and citations. The country is home to institutions leading in both publication and citation metrics. Prominent authors contributing significantly to this domain include Conwell Yeates, Van Orden Kimberly A., and Waern Margda. Key journals disseminating crucial research are the Journal of Affective Disorders, American Journal of Geriatric Psychiatry, and Lancet. Frequent keywords in this field encompass depression, suicidal ideation, suicide, older-adults, risk, risk factors, prevalence, older adults, ideation, behavior, health, mental health, life, age, people, prevention, symptoms, scale, population, and elderly. The contemporary research emphasis is primarily on identifying, treating, and preventing the suicide risk associated with depression in older adults.

**Conclusions:**

This study highlights the growing research focus on suicide in older adults, particularly related to depression and the identification, treatment, and prevention of suicide risk. The findings underscore the need for targeted prevention strategies and further investigation in this critical public health area.

## 1 Introduction

According to a World Population Prospect (1), by 2030, one in six people worldwide will be aged 60 or older. By 2050, the number of individuals aged 60 and above will double from 2020 levels, reaching 2.1 billion. It is projected that between 2020 and 2050, the population of those aged 80 and above will triple, reaching 426 million. Meanwhile, suicide remains a serious public health issue, causing approximately 700,000 deaths globally each year (2). Of these deaths, around one quarter (27.2%) occur among individuals aged 60 and above (3).

Klonsky et al. (4) proposed the ideation-to-action Three-Step Theory, which outlines the progression from suicidal ideation to strong ideation and ultimately a suicide attempt. Previous research (5) has identified female gender, history of smoking, lack of income, depressive symptoms, and heart disease as predictors of suicidal ideation in the elderly. Among older adults with suicidal ideation, 6% to 20% attempt suicide (6). According to the life course development theory (7), as elderly individuals experience a decline in physical function and reduced opportunities for life control, those who fail to adapt to these changes may face a higher risk of suicide. Elderly individuals who attempt suicide exhibit higher suicidal intent and greater medical lethality, with disease-related issues strongly motivating their suicidal behavior (8). Studies have found that over 90% of those who complete suicide are diagnosed with severe mental illness, and 50% suffer from depression at the time of their suicide (9). Depression is a major risk factor for suicide (10–13). A Swedish cohort study found that the risk of suicide in individuals with depression is more than 15 times higher compared to the general population. Notably, during the first three months following a diagnosis, this risk increases to 32 times higher (14). This elevated risk is even more pronounced in the elderly population (15). Additionally, a history of previous suicide attempts in individuals with depression further indicates a higher likelihood of future suicide risk (16). Furthermore, the severity of depression is strongly associated with suicide risk (11, 17, 18). Elderly individuals with moderate to severe depression, as measured by the eight-item Patient Health Questionnaire depression scale (PHQ-8≥15), are 48 times more likely to experience suicidal ideation compared to those with minimal or mild depressive symptoms (PHQ-8 <10) (17).

Cognitive impairment is commonly observed among elderly individuals who commit suicide. Late-stage dementia typically does not accompany an increased risk of suicide, as the decline in cognitive abilities prevents patients from recognizing their condition and limits their behavioral choices (19). However, patients with early-stage dementia often experience distress due to their awareness of cognitive changes and fear of future limitations, which may increase their suicide risk (19). Previous study (19) has indicated that individuals with mild cognitive impairment or early-stage dementia are at an increased risk of attempted suicide, particularly within the first 90 days and the first year following diagnosis. Additionally, executive dysfunction is associated with a sevenfold increase in the risk of suicide (20). However, it does not significantly impact suicidal ideation or suicide attempts (20).

Bibliometrics employs qualitative and quantitative methods to analyze, assess, and manage literature and information resources, integrating techniques from information science, statistics, and computer science to measure the productivity, impact, and trends of scientific research (21). Bibliometrics involves the analysis of various indicators, including citation analysis, author collaboration networks, international research partnerships, institutional collaborations, journal citation patterns, and the evolution of research themes. This field aids researchers and policymakers in understanding the dynamic landscapes of academic disciplines, thereby facilitating the development of research strategies and the allocation of resources. Currently, suicide among the elderly is primarily associated with depression, and significant research findings have emerged over the past 20 years; however, bibliometric analyses in this area remain sparse. This study seeks to undertake a comprehensive bibliometric analysis of research related to suicide in the elderly, employing advanced bibliometric software tools including VOSviewer, Pajek, and CiteSpace. The goal is to uncover trends and emerging themes from the last two decades, facilitating a deeper understanding of the evolving landscape within this scholarly domain. The insights gained from this analysis will be instrumental for researchers and policymakers in shaping strategic research initiatives and optimizing resource allocation.

## 2 Materials and methods

### 2.1 Search strategy and data retrieval

The data for the bibliometric analysis conducted in this study were obtained from the Web of Science Core Collection (WOSCC). Recognized for its comprehensiveness and standardization, WOSCC is extensively used in academic research (22). Our retrieval methodology employs “TS=(“old* people” OR “old* adult*” OR “old* person” OR “old* population” OR “elder* people” OR “elder* adult*” OR “elder* population” OR “aged people” OR geriatric* OR Centenarians OR Nonagenarians OR Octogenarians)” AND TS=(“Suicid* Ideation” OR “Suicid* Assisted” OR “Suicid* Attempt*” OR “Suicid* Completed” OR “Suicid* Prevention”). The latest update to the search was performed on July 2, 2024, encompassing publications from January 1, 2005, to July 2, 2024. A total of 1,116 English-language documents, classified solely as “articles” and “reviews,” were selected for analysis. These documents were subsequently exported in plain text format for in-depth analysis.

### 2.2 Statistical analysis and visualization of data

For comprehensive visual analyses, we employed several tools: CiteSpace 6.3.R3 Advanced by Chaomei Chen (23), VOSviewer by Nees Jan van Eck and colleagues (24), and Pajek software by Batagelj, V et al. (25). Key information such as titles, authors, affiliations, countries or regions, publication journals, keywords, and references were extracted to visualize collaboration networks among countries, institutions, and authors. We also created keyword co-occurrence networks, keyword timelines, and conducted co-citation analyses for authors, journals, and references. Data aggregation, organization, and visualization were performed using Microsoft 365 Excel. As the data used in this study were sourced from public databases, no ethical review was necessary.

## 3 Results

### 3.1 Retrieval diagram, annual publications, and citations

**Figure 1:**
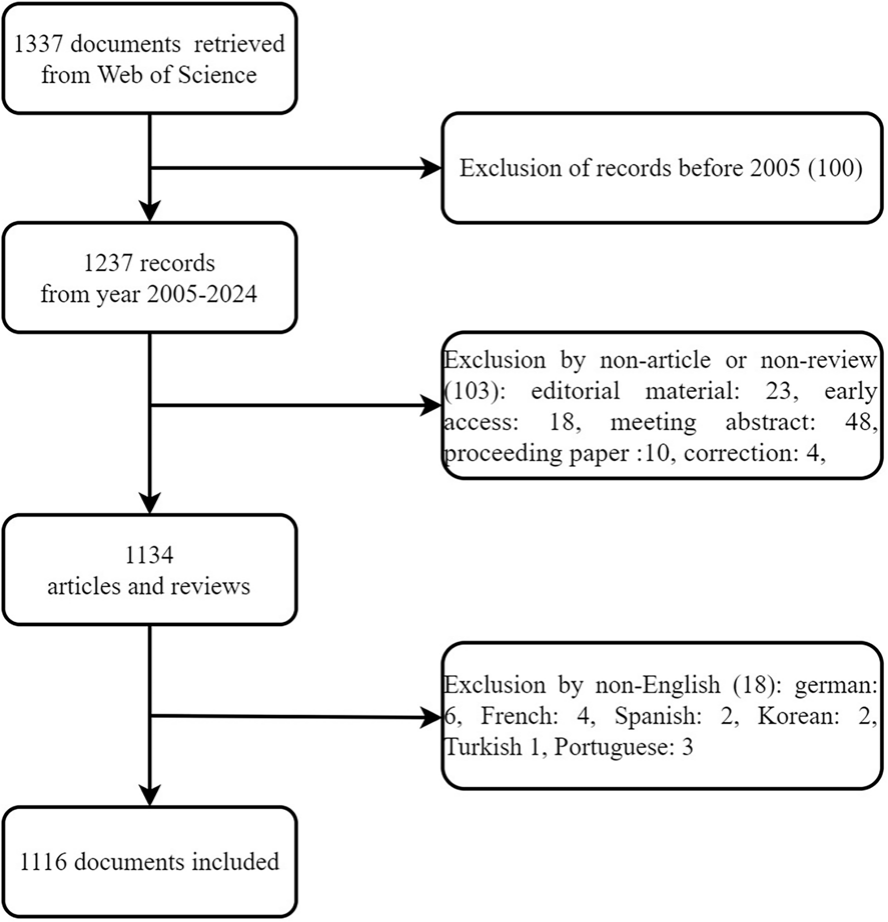
Flow diagram.

**Figure 2:**
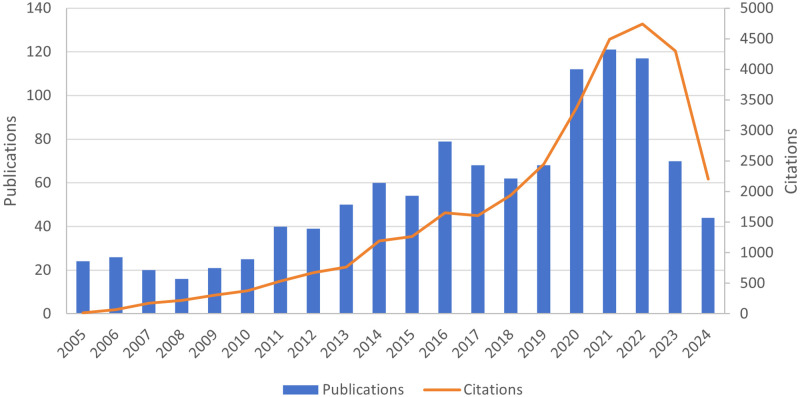
The annual number of publications and citations of relevant articles from 2005 to 2024, with data for 2024 updated until July 2.

Between 2005 and 2024, our search yielded 1,337 records, all of which were English-language articles and reviews. After a thorough screening process, 1,116 documents were included in the final analysis (**Figure 1**). **Figure 2** illustrates the annual publication trends and citation frequencies of articles concerning suicide in elderly adults over the past 20 years. The annual number of publications exhibited a fluctuating upward trend. Notable decreases were observed in 2007, 2012, 2015, 2017, and 2022, followed by increases in 2009, 2013, 2016, and 2019. Since 2022, the trend has been declining. The annual publication count peaked at 121 in 2021, while citation frequencies reached their highest point of 4,741 in 2022.

### 3.2 Distribution of countries or regions

Research on suicide in the elderly people encompasses contributions from 56 countries or regions. **Table 1** showcases the top 10 countries or regions, ranked by publication volume, citation frequency, and average citations per article. The United States (USA) leads in publication volume with 528 articles, followed by China with 146 articles. South Korea and Canada come next with 122 and 113 articles, respectively, while all other countries and regions have fewer than 100 publications each. In terms of citation frequency, the USA again ranks first with 18,514 citations, followed by the United Kingdom with 5,947 citations, Canada with 3,843 citations, and Australia with 3,307 citations. All remaining countries and regions have fewer than 3,000 citations each. Belgium leads with the highest average citations per article at 109.93, followed by Denmark at 65.50, Germany at 63.88, the United Kingdom at 61.95, Italy at 55.10, and Uganda at 50.67. Average citations per article for all other countries or regions are below 50.

**Table 1:** Top 10 Countries or Regions by publication count and citation frequency.

Rank	Countries/Regions	Publications	Country/Regions	Citations	Country/Regions	Citations per article
1	USA	528	USA	18514	Belgium	109.93
2	China	146	United Kingdom	5947	Denmark	65.50
3	South Korea	122	Canada	3843	Germany	63.88
4	Canada	113	Australia	3307	United Kingdom	61.95
5	Australia	97	China	2080	Italy	55.10
6	United Kingdom	96	South Korea	1722	Uganda	50.67
7	France	39	Belgium	1649	New Zealand	40.53
8	Sweden	39	Italy	1598	Singapore	37.50
9	Spain	33	Sweden	1450	Sweden	37.18
10	Italy	29	France	1228	USA	35.06

**Figure 3:**
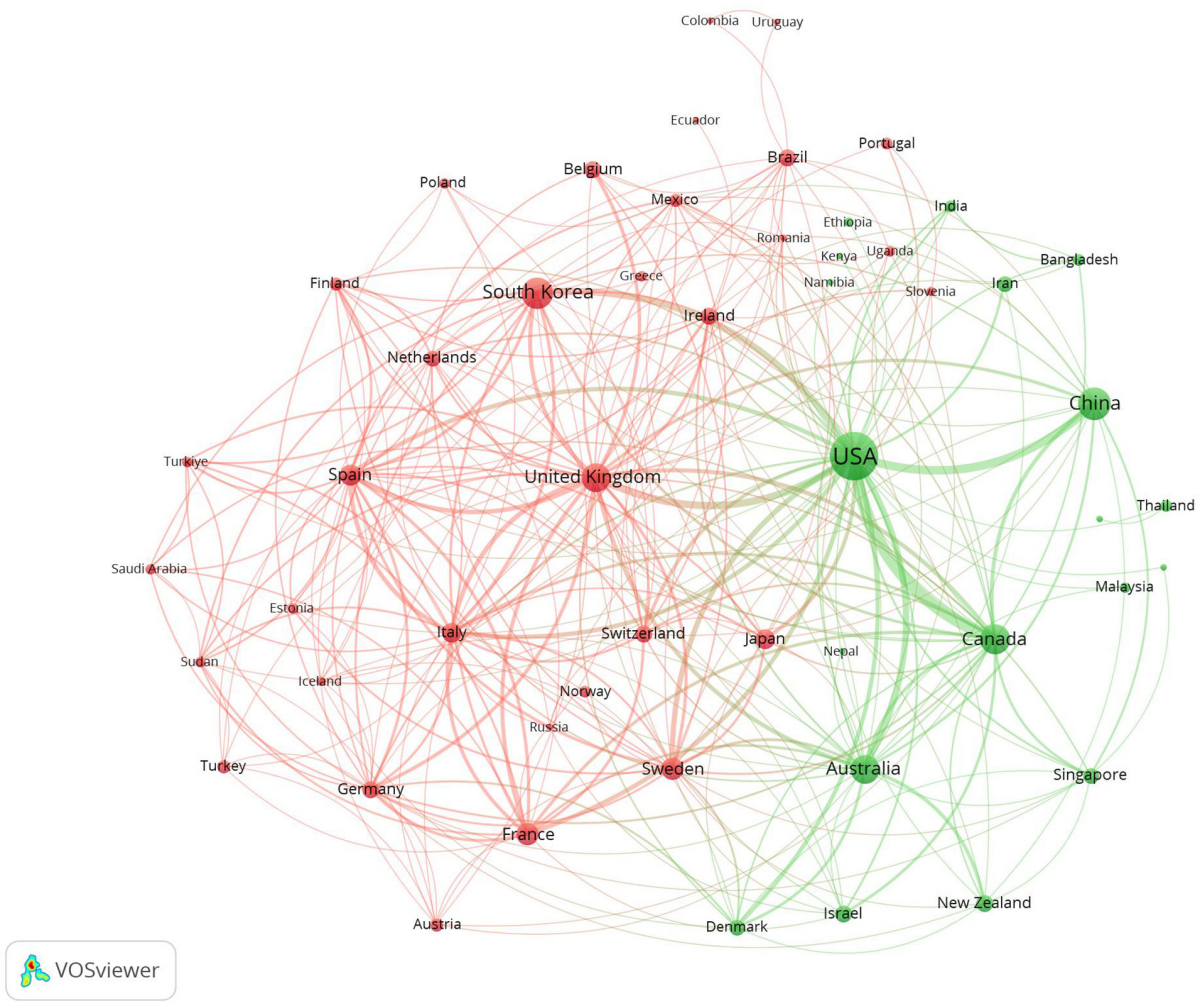
Collaboration networks among countries/regions were visualized using VOSviewer. Nodes are color-coded to represent distinct clusters, with node size indicating the relative prominence of each country/region.

**Figure 3** visualizes the collaborative dynamics among countries and regions engaged in research on suicide in the elderly using VOSviewer. Countries and regions are clustered based on the intensity of their collaboration, depicted in different colors. For instance, the red cluster includes South Korea, United Kingdom, France, Sweden, Spain, Italy, and others, while the green cluster encompasses the USA, China, Canada, Australia, Israel, New Zealand, and additional countries. Node sizes correspond to publication volumes, with larger nodes representing countries like the USA, China, United Kingdom, Canada, France, and Australia. The thickness of connecting lines indicates the strength of inter-country collaborations. The results highlight strong partnerships, particularly between the USA and countries such as China, Australia, and Canada, emphasizing their significant roles in advancing research on suicide in the elderly.

### 3.3 Distribution of institutions

Research on suicide among the elderly involves 1,492 affiliations. **Table 2** highlights the top 10 affiliations based on publication frequency and citation impact. The University of Rochester leads with 97 publications, followed by the University of Pittsburgh with 55, the University of Toronto with 34, and Shandong University with 30. Other institutions each have fewer than 30 publications. In terms of citation impact, the University of Rochester also stands out with 5,018 citations, followed by the University of Pittsburgh with 2,415 citations, Cornell University with 1,858, Florida State University with 1,605, Warneford Hospital with 1,566, and Texas Tech University with 1,525. The remaining institutions each have fewer than 1,500 citations.

**Table 2:** The top 10 institutions by publication volume and citation frequency.

Rank	Institutions	Publications	Institutions	Citations
1	University of Rochester (USA)	97	University of Rochester (USA)	5018
2	University of Pittsburgh (USA)	55	University of Pittsburgh (USA)	2415
3	University of Toronto (Canada)	34	Cornell University (USA)	1858
4	Shandong University (China)	30	Florida State University (USA)	1605
5	University of Pennsylvania (USA)	28	Warneford Hospital (UK)	1566
6	University of Washington (USA)	26	Texas Tech University (USA)	1525
7	University of Hong Kong (China)	25	Ghent University Hospital (Belgium)	1424
8	University of Michigan (USA)	25	University of Pennsylvania (USA)	1309
9	University of Gothenburg (Sweden)	24	University of Toronto (Canada)	1297
10	Columbia University (USA)	23	Weill Cornell Institute of Geriatric Psychiatry (USA)	1198

**Figure 4:**
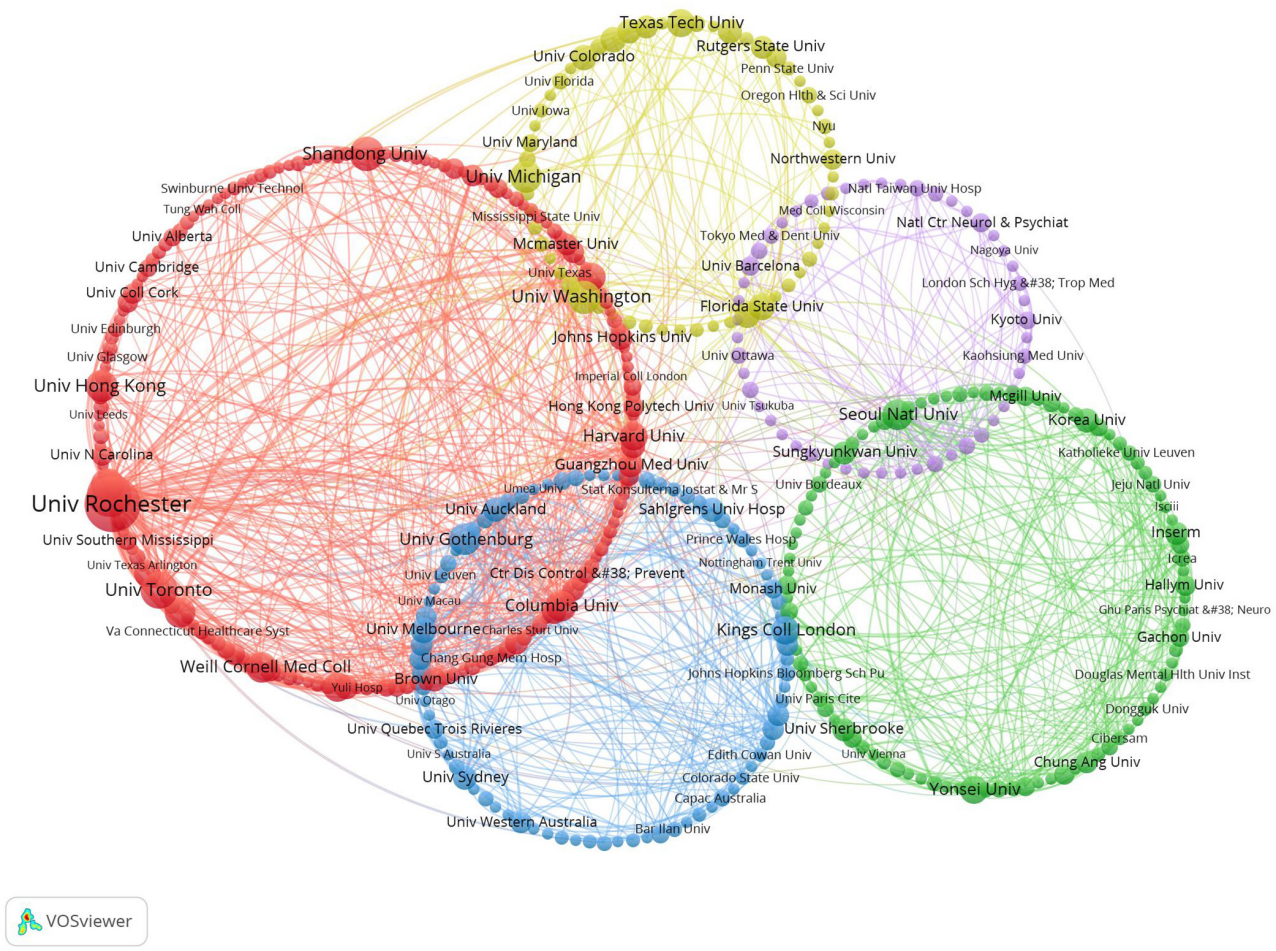
Collaboration networks among institutions were visualized using VOSviewer and Pajek. Nodes of different colors represent distinct institutional clusters, and the size of each node reflects the frequency of occurrence.

**Figure 4** visually represents the collaborative network among institutions engaged in research on suicide in the elderly. Utilizing VOSviewer, the analysis categorizes these institutions into five distinct circles, each represented by a different color to indicate the intensity of their collaborative relationships. The red cluster encompasses renowned institutions such as the University of Rochester, University of Pittsburgh, University of Toronto, and University of Western Ontario. In contrast, the green cluster includes Seoul National University, Yonsei University, Anglia Ruskin University, and Bezmialem Vakif University. Within the blue cluster are prominent institutions like the University of Gothenburg, University of Melbourne, King’s College London, and the University of Sydney. The yellow cluster features esteemed universities such as the University of Washington, Duke University, University of Michigan, and the University of California, Los Angeles. Lastly, the purple cluster comprises the University of Tokyo, National Taiwan University Hospital, National Center of Neurology and Psychiatry, and Emory University.

These institutions serve as central hubs within their respective clusters, demonstrating robust collaborative connections. Node sizes reflect publication volumes, highlighting significant contributors like the University of Rochester, Seoul National University, University of Gothenburg, University of Tokyo, and University of Washington, which have made substantial impacts in this field of research.

### 3.4 Distribution of authors

Examining the authors who have made significant contributions to research on suicide in the elderly reveals both the prominent figures and prevailing trends in this field. A total of 4119 authors have participated in studies related to this topic. **Table 3** highlights the top 10 authors based on their publication records and citation frequencies. Conwell Yeates leads with 42 publications, followed by Waern Margda26, Szanto Katalin25, Van Orden Kimberly A with 22 publications, and Heisel Marnin J with 21 publications. All other authors have published fewer than 20 papers. In terms of citation frequencies, Van Orden Kimberly A holds the top position with 1778 citations, followed by Conwell Yeates with 1766 citations and Hawton Keith with 1661 citations. The remaining authors have been cited fewer than 1500 times each.

**Table 3:** The top 10 authors by publication volume and citation frequency.

Rank	Author	Publications	Author	Citations
1	Conwell, Yeates	42	Van Orden, Kimberly A.	1778
2	Waern, Margda	26	Conwell, Yeates	1766
3	Szanto, Katalin	25	Hawton, Keith	1661
4	Van Orden, Kimberly A.	22	Cukrowicz, Kelly C.	1421
5	Heisel, Marnin J.	21	Szanto, Katalin	1232
6	Reynolds, Charles F., Iii	17	Joiner, Thomas E., Jr.	1201
7	Dombrovski, Alexandre Y.	16	Waern, Margda	1106
8	Bruce, Martha L.	15	Witte, Tracy K.	1032
9	Duberstein, Paul R.	15	Erlangsen, Annette	943
10	Choi, Namkee G.	14	O’Connor, Rory C.	872

**Figure 5:**
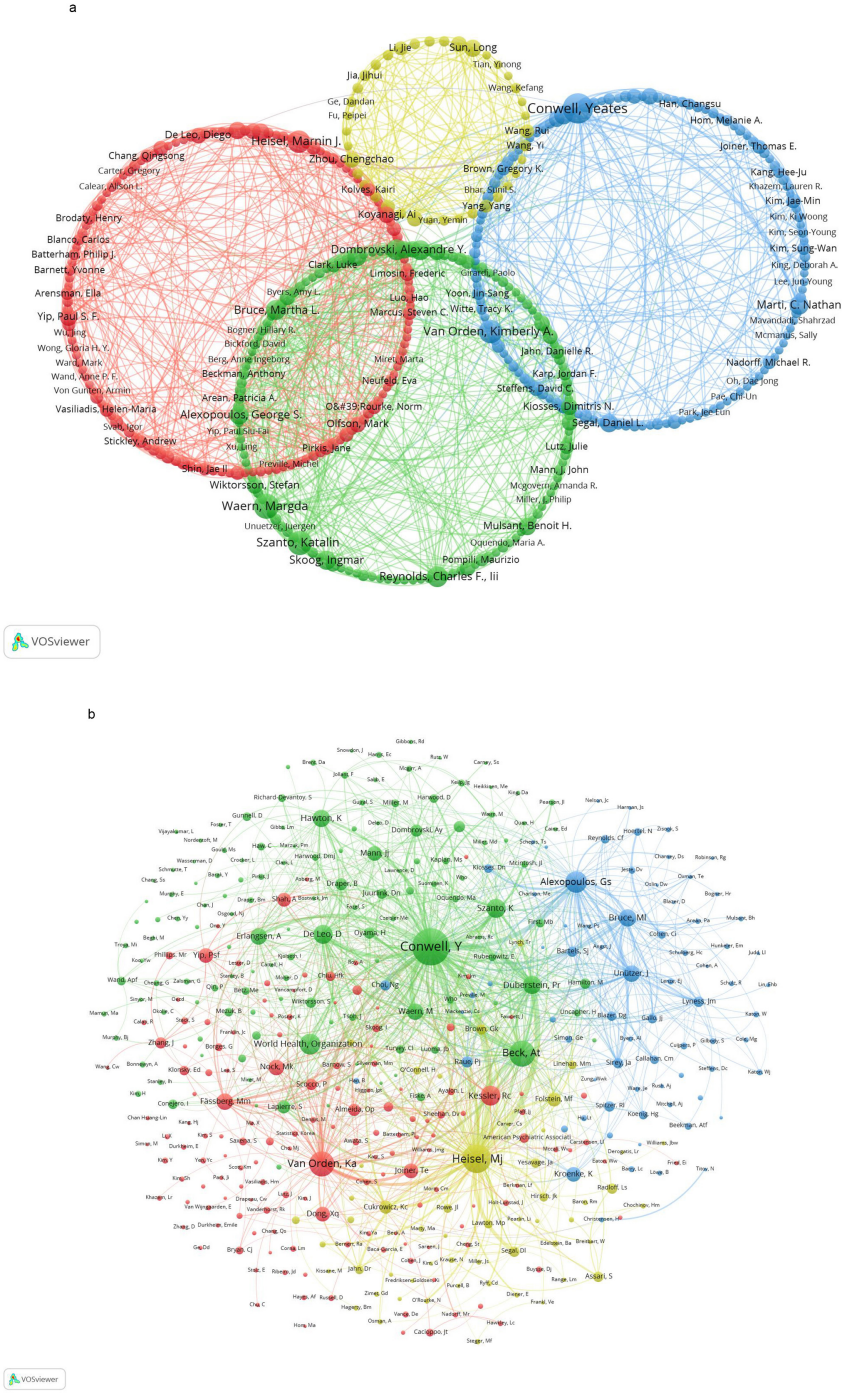
Analysis of authors related to suicide in older adults. **(A)** Collaboration networks among authors were visualized using VOSviewer and Pajek. Nodes of different colors represent authors within distinct clusters, and node size corresponds to the frequency of occurrence. **(B)** Citation networks among authors were visualized using VOSviewer, with node size indicating the frequency of occurrence.

**Figure 5A** visually maps the collaborative network among authors engaged in research on suicide in the elderly. Using VOSviewer, authors are categorized into four distinct clusters based on their collaborative strength, each marked by a unique color. The red cluster features prominent authors such as Draper Brian, Koyanagi Ai, and Heisel Marnin J. The green cluster includes Waern Margda, Szanto Katalin, and Reynolds Charles F III. In the blue cluster, notable authors include Conwell Yeates, Van Orden Kimberly A, and Kim Jae-Min. The size of each node corresponds to the publication volume of the respective authors, while the thickness of the connecting lines indicates the strength of their collaborative relationships. Notably, authors with significant publication volumes include Conwell Yeates, Marti C Nathan, Bruce Martha L, and Heisel Marnin J.

**Figure 5B** illustrates the co-citation network among authors in the field, analyzed using VOSviewer. Authors are grouped into four primary clusters based on the strength of their co-citation relationships, each represented by a unique color. The red cluster includes prominent authors such as Van Orden KA, Kessler RC, and Joiner TE. In the green cluster, notable figures include Conwell Y, Beck AT, and Duberstein PR. The blue cluster comprises authors like Alexopoulos GS, Bruce ML, and Raue PJ. The yellow cluster encompasses authors such as Heisel MJ, Cukrowicz KC, and Folstein MF. Each cluster’s size corresponds to the frequency of co-citations among its authors, with larger circles indicating stronger co-citation weights. These authors are among the most frequently cited within their respective clusters.

### 3.5 Distribution of journals

Research on elderly related to suicide is published across 323 journals, detailed in **Table 4** which ranks the top 10 journals by both publication volume and citation frequency. Leading journals in terms of publications include Journal of Affective Disorders with 71 articles, American Journal of Geriatric Psychiatry with 70 articles, Aging & Mental Health with 69 articles, and International Journal of Geriatric Psychiatry with 52 articles. Other journals have published fewer than 50 articles each. The most cited journals include Lancet with 2584 citations, American Journal of Geriatric Psychiatry with 2319 citations, and Journal of Affective Disorders with 2232 citations, while other journals have citation frequencies below 2000.

**Table 4:** The top 10 journals by publication volume and citation frequency.

Rank	Journals	Publications	Journals	Co-citations
1	Journal of Affective Disorders	71	Lancet	2584
2	American Journal of Geriatric Psychiatry	70	American Journal of Geriatric Psychiatry	2319
3	Aging & Mental Health	69	Journal of Affective Disorders	2232
4	International Journal of Geriatric Psychiatry	52	Aging & Mental Health	1855
5	International Psychogeriatrics	47	International Journal of Geriatric Psychiatry	1164
6	Clinical Gerontologist	24	British Journal of Psychiatry	1104
7	International Journal of Environmental Research and Public Health	21	Psychological Assessment	947
8	Psychiatry Research	21	International Psychogeriatrics	877
9	Frontiers in Psychiatry	18	Psychiatry Research	830
10	Suicide and Life-Threatening Behavior	18	BMC Psychiatry	773

**Figure 6:**
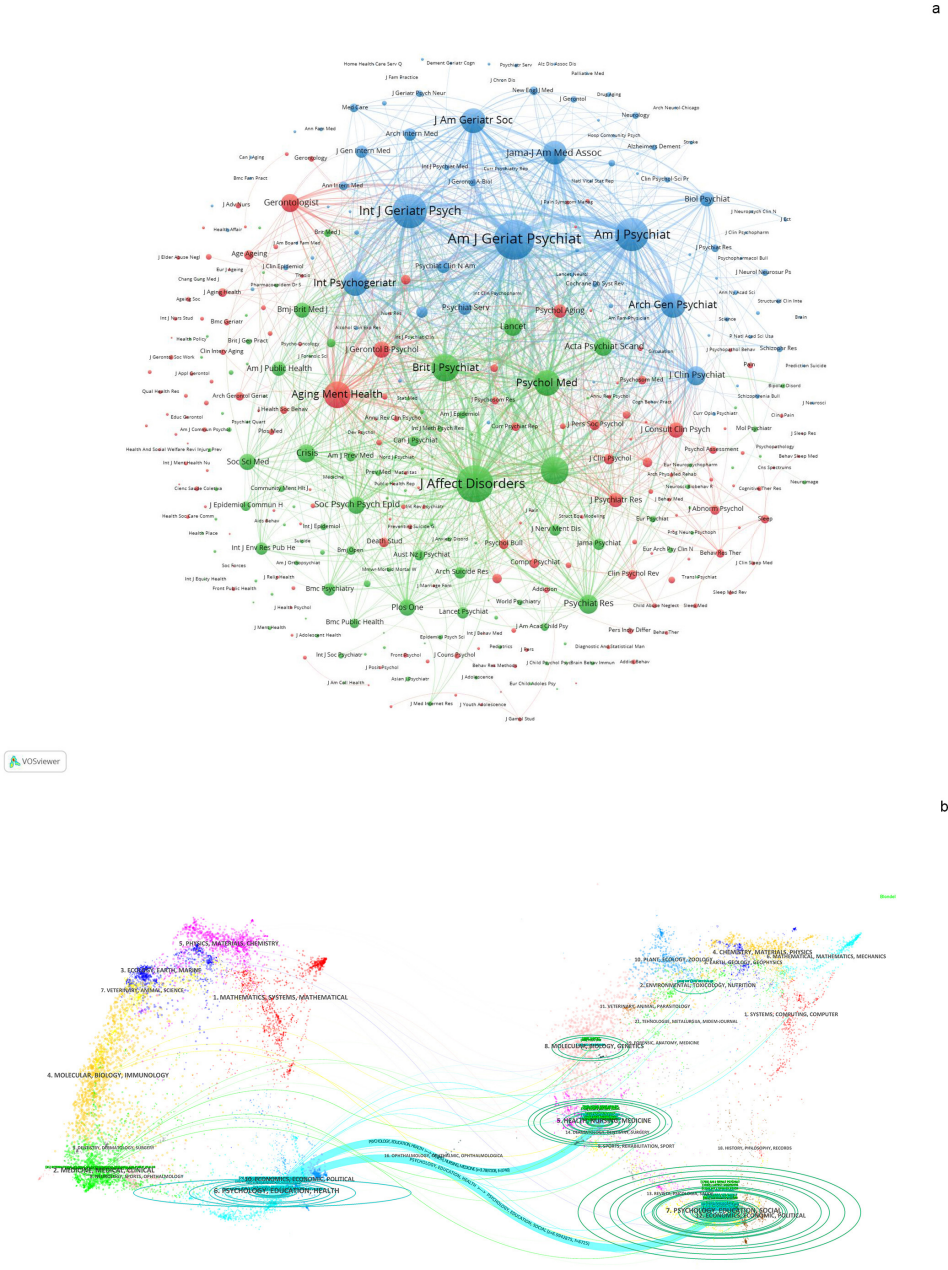
Analysis of authors and journals related to suicide in older adults. **(A)** Collaboration networks among journals were visualized using VOSviewer. Nodes of different colors represent journals within distinct clusters, and node size reflects their frequency of occurrence. **(B)** A dual-map overlay of journals is presented, with citing journals on the left and cited journals on the right. Colored pathways illustrate citation relationships.

**Figure 6A** illustrates the network of journals that publish research articles on suicide-related elderly, analyzed using VOSviewer to visualize their interconnections. These journals are grouped into 3 distinct clusters identified by different colors, which represent their thematic similarities. The red cluster includes journals such as Aging & Mental Health, The Gerontologist, and The Journals of Gerontology Series B: Psychological Sciences. In the green cluster, you find Journal of Affective Disorders, Suicide and Life-Threatening Behavior, and British Journal of Psychiatry. The blue cluster encompasses American Journal of Geriatric Psychiatry, American Journal of Psychiatry, and International Journal of Geriatric Psychiatry.

Knowledge flow analysis was utilized to explore the evolution of citation and co-citation patterns among journals, examining how they influence each other over time (26). The dual-map overlay of journals provides a visual representation of how topics are disseminated, citation pathways are established, and research focal points shift across academic literature (26, 27). On the left side of the Dual-map, labels represent journals citing others, while those on the right denote journals being cited. The colored curves connecting the citing map to the cited map visually depict the comprehensive context of each citation. In the citing map, the vertical axis of the ellipse expands with an increasing number of papers published by a journal, while the horizontal axis expands corresponding to a larger number of authors involved. Citing journals predominantly cover topics in fields such as PSYCHOLOGY, EDUCATION, HEALTH, MEDICINE, MEDICAL, and CLINICAL, representing the forefront of research. The topics addressed by cited journals mainly encompass areas like PSYCHOLOGY, EDUCATION, SOCIAL, HEALTH, NURSING, MEDICINE, MOLECULAR, BIOLOGY, GENETICS, ENVIRONMENTAL, TOXICOLOGY, NUTRITION, forming the foundational knowledge base in **Figure 6B**.

### 3.6 Analysis of keywords

**Figure 7:**
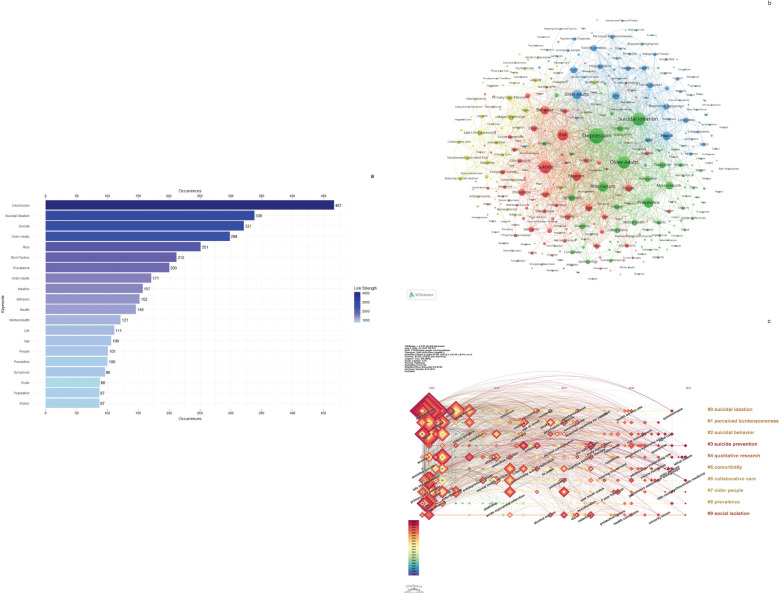
**(A)** The top 20 most frequently occurring keywords and their link strengths. **(B, C)** Analysis of keywords related to suicide in older adults. **(B)** Keyword co-occurrence networks were visualized using VOSviewer, with nodes in different colors representing distinct keyword clusters. Node size reflects their frequency of occurrence. **(C)** A visual timeline created with CiteSpace depicts the evolutionary emergence of these keywords, with the size of each square indicating their frequency over time.

Keywords play a pivotal role in navigating the cutting-edge research landscape of suicide-related elderly. **Figure 7A** highlights the top 20 frequently cited keywords, providing a comprehensive glimpse into the forefront of this field. Leading the list is depression, followed by suicidal ideation, suicide, older-adults, risk, risk-factors, prevalence, older adults, ideation, behavior, health, mental-health, life, age, people, prevention, symptoms, scale, population, and elderly. The top 20 keywords are mentioned between 87 and 467 times, whereas all other keywords appear less frequently, each below 87 occurrences.

**Figure 7B** depicts a network of keyword co-occurrences, where closely related terms are organized into 4 distinct clusters distinguished by different colors. The red cluster encompasses terms such as suicide, risk, ideation, behavior, and age. In the green cluster, you find keywords like depression, suicidal ideation, older adults, risk factors, and prevalence. The blue cluster includes terms such as older adults, health, life, and scale. The yellow cluster comprises major depression, late-life depression, and primary care patients.

**Figure 7C** provides a visual timeline depicting the evolutionary emergence of these keywords, where the size of each square corresponds to how frequently it appears over time. The keywords listed in the right column are aligned with the thematic clusters observed on the left.

### 3.7 Analysis of reference

**Table 5** presents the top 15 most frequently cited articles, with the leading article (28), titled “Suicide” published in the Lancet, having been cited 1,413 times. This article provides a comprehensive analysis of the global landscape of suicide and prevention strategies. It highlights the significant prevalence of psychiatric disorders among those who die by suicide and underscores the numerous challenges faced in implementing effective suicide prevention measures, particularly in developing countries. The article (29) titled “Depression in the Elderly,” which appears in the Lancet, ranks second in citation frequency with a total of 1,171 citations. This article examines the prevalence and impact of depression among the elderly, focusing on its association with chronic medical conditions, cognitive impairment, and psychosocial stressors. It also evaluates the effectiveness of antidepressant treatments and highlights the inadequacies of public insurance coverage for these services in North America. The remaining articles in the top 15 by citation frequency have each garnered fewer than 1,000 citations.

**Table 5:** The top 15 articles by citation frequency.

Rank	Article title	Authors	Source title	Year	Citations	DOI	Document Type
1	Suicide	Hawton (28).	Lancet	2009	1413	10.1016/S0140-6736(09)60372-X	Review
2	Depression in the elderly	Alexopoulos, GS et al. (29)	Lancet	2005	1171	10.1016/S0140-6736(05)66665-2	Review
3	Thwarted Belongingness and Perceived Burdensomeness: Construct Validity and Psychometric Properties of the Interpersonal Needs Questionnaire	Van Orden, KA et al. (43)	Psychological Assessment	2012	900	10.1037/a0025358	Article
4	Mental health and well-being during the COVID-19 pandemic: longitudinal analyses of adults in the UK COVID-19 Mental Health & Wellbeing study	O’Connor, RC et al. (44)	British Journal of Psychiatry	2021	724	10.1192/bjp.2020.212	Article
5	Loneliness in the general population: prevalence, determinants and relations to mental health	Beutel, ME et al. (45)	BMC Psychiatry	2017	616	10.1186/s12888-017-1262-x	Article
6	Mental health of prisoners: prevalence, adverse outcomes, and interventions	Fazel, S et al. (46)	Lancet Psychiatry	2016	553	10.1016/S2215-0366(16)30142-0	Review
7	A tune in a minor can b major: A review of epidemiology, illness course, and public health implications of subthreshold depression in older adults	Meeks, TW et al. (47)	Journal of Affective Disorders	2011	363	10.1016/j.jad.2010.09.015	Review
8	Suicidal thoughts and behaviors and social isolation: A narrative review of the literature	Calati, R et al. (48)	Journal of Affective Disorders	2019	330	10.1016/j.jad.2018.11.022	Review
9	Evaluating factors and interventions that influence help-seeking and mental health service utilization among suicidal individuals: A review of the literature	Hom, MA et al. (49)	Clinical Psychology Review	2015	279	10.1016/j.cpr.2015.05.006	Article
10	Dialectical behavior therapy skills use as a mediator and outcome of treatment for borderline personality disorder	Neacsiu, AD et al. (50)	Behaviour Research and Therapy	2010	259	10.1016/j.brat.2010.05.017	Article
11	Psychometric properties of the 15-item Geriatric Depression Scale in functionally impaired, cognitively intact, community-dwelling elderly primary care patients	Friedman, B et al. (51)	Journal of the American Geriatrics Society	2005	248	10.1111/j.1532-5415.2005.53461.x	Article
12	A systematic review of physical illness, functional disability, and suicidal behaviour among older adults	Fässberg, MM et al. (52)	Aging & Mental Health	2016	239	10.1080/13607863.2015.1083945	Review
13	Psychological resilience in young and older adults	Gooding, PA et al. (53)	International Journal of Geriatric Psychiatry	2012	233	10.1002/gps.2712	Article
14	A Systematic Review of Social Factors and Suicidal Behavior in Older Adulthood	Fässberg, MM et al. (54)	International Journal of Environmental Research and Public Health	2012	219	10.3390/ijerph9030722	Review
15	Suicide in older adults: current perspectives	Conejero, I et al. (42)	Clinical Interventions in Aging	2018	213	10.2147/CIA.S130670	Review

**Figure 8:**
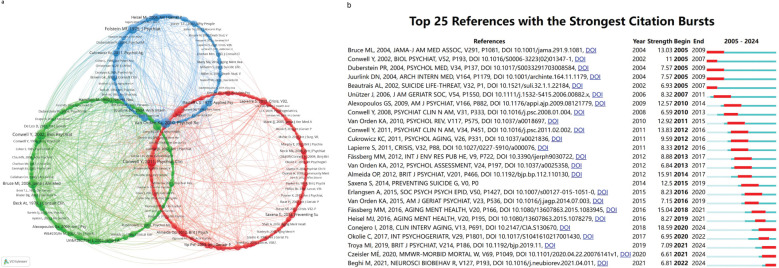
**(A)** Citation networks among references were visualized using VOSviewer and Pajek, with node size representing the frequency of occurrence. **(B)** The top 25 references exhibiting the strongest citation bursts.

Reference co-citation analysis, which examines the interrelationships among references based on their shared citation frequencies, was utilized to identify citation patterns. **Figure 8A** displays a relational diagram that illustrates these connections among studies, organized into three main clusters, each distinguished by unique colors. In the red cluster, foundational references include Conwell Y 2011 (30), Lapierre S 2011 (31), and Almeida OP 2012 (32). The green cluster features references such as Conwell Y 2002 (33), Bruce ML 2004 (34), and Beck AT 1979 (35). The blue cluster comprises references including Van Orden KA 2010 (36), Folstein MF 1975 (37), and Heisel MJ 2006 (38). The figure demonstrates that the size of each circle reflects its co-citation weight, representing the relative importance of these references within their respective color-coded clusters.

**Figure 8B** illustrates the top 25 references with significant citation bursts, with 2005 standing out as both the year with the highest number of citation bursts, totaling five, and the year that saw the earliest burst. Notably featured is the study by Bruce ML et al. (34), titled “Reducing Suicidal Ideation and Depressive Symptoms in Depressed Older Primary Care Patients: A Randomized Controlled Trial,” published in 2004 in JAMA. This study evaluates the Prevention of Suicide in Primary Care Elderly: Collaborative Trial (PROSPECT), a randomized controlled trial that assessed the impact of a tailored primary care intervention on suicidal ideation and depression among older adults. It found that the intervention significantly reduced suicidal ideation and improved depression outcomes compared to usual care, highlighting its potential as an effective prevention strategy in late life. In 2005, four other significant studies also experienced notable citation bursts. These include the study by Conwell et al. (33) from 2002, titled “Risk Factors for Suicide in Later Life”; the research by Duberstein et al. (39) from 2004, “Suicide at 50 Years of Age and Older: Perceived Physical Illness, Family Discord, and Financial Strain”; the 2004 paper by Juurlink et al. (40), “Medical Illness and the Risk of Suicide in the Elderly”; and the 2002 article by Beautrais AL et al. (41), “A Case-Control Study of Suicide and Attempted Suicide in Older Adults.” These studies collectively highlight critical insights into suicide risk among older adults. They identify affective disorders, physical illnesses, and psychosocial factors—such as family discord and limited social networks—as significant contributors to suicide risk. Effective prevention strategies should focus on improving detection and treatment of mood disorders, addressing severe physical illness, and mitigating financial and social stressors. The research underscores the importance of tailored interventions and the need for further studies to refine understanding of these risk factors. The paper by Conejero I et al (42), titled “Suicide in Older Adults: Current Perspectives,” achieved the highest citation burst intensity of 18.59 in 2018. This narrative review evaluates recent findings on suicide risk factors in older adults, emphasizing the roles of psychiatric and neurocognitive disorders, social exclusion, and chronic physical illnesses. It underscores the necessity of integrating these factors into prevention models and adapting chronic care approaches to better address depression and suicidal behaviors in older populations.

## 4 Discussion

Our bibliometric analysis of suicide in the elderly employs VOSviewer, Pajek, and CiteSpace, examining publications from January 1, 2005, to July 2, 2024. This study includes contributions from 56 countries or regions, 1,492 institutions, and 4,119 authors. Our findings indicate that publication output peaked at 121 papers in 2021, while citation frequency reached its highest point of 4,741 in 2022 (**Figure 2**). The peak in publication volume and citation frequency aligns with the significant increase in suicide rates among individuals aged 65 and older in 2022 (55). Subsequently, both publication numbers and citation rates have experienced varying degrees of decline in the following years. This may be related to the implementation of effective suicide intervention measures in several countries, such as the launch of the 988 Suicide & Crisis Lifeline by the U.S. Department of Health and Human Services’ Substance Abuse and Mental Health Services Administration (SAMHSA).

The analysis of countries or regions reveals that in the field of suicide in the elderly, the USA significantly outpaces other nations in both publication volume and citation frequency, establishing itself as the most influential country in this domain (**Table 1**). Additionally, among the top 10 institutions by publication volume, 6 are based in the USA, and among the top 10 institutions by citation frequency, 7 are also from the USA (**Table 2**). The University of Rochester ranks first in both publication volume with 97 papers and citation frequency with 5018 citations, highlighting its predominant impact in the field of suicide in elderly. In terms of collaboration, the USA occupies a central position with the highest and strongest connections. Furthermore, countries with higher publication volumes such as China, United Kingdom, South Korea, and Canada also maintain close exchanges and collaborations with other nations and regions (**Figure 3**). Suicide is a significant public health issue; however, the quality of global data on suicide and self-harm remains suboptimal. This is partly due to the stigma surrounding suicide in many societies and the criminalization of suicidal behavior in certain countries. Moreover, variations in suicide rates and methods across different nations present additional challenges. Research on this topic not only requires substantial human and material resources but also necessitates support at the national level. For instance, in many Arab countries, suicide data is not disclosed due to religious prohibitions. The USA, as a developed country and one of the pioneers in establishing suicide prevention mechanisms, plays a critical role in research on suicide among the elderly globally.

Conwell Yeates is a leading figure in the field of suicide research among the elderly, holding the top position in publication volume and ranking second in citation frequency (**Table 3**). Notably, Hawton K et al.’s 2009 study (28) titled “Suicide,” published in the Lancet, has achieved the highest citation frequency. Additionally, Van Orden KA et al.’s 2012 research (43), “Thwarted Belongingness and Perceived Burdensomeness: Construct Validity and Psychometric Properties of the Interpersonal Needs Questionnaire,” published in Psychological Assessment, ranks third in citation frequency. O’Connor RC et al.’s 2021 study (44), “Mental Health and Well-being During the COVID-19 Pandemic: Longitudinal Analyses of Adults in the UK COVID-19 Mental Health & Wellbeing Study,” published in the British Journal of Psychiatry, follows closely, ranking fourth in citation frequency.

In our comprehensive analysis of journals, we identified six that stand out not only for their high publication volume but also for their significant citation frequency. These journals, which are ranked within the top ten in both categories, include the Journal of Affective Disorders, the American Journal of Geriatric Psychiatry, Aging & Mental Health, the International Journal of Geriatric Psychiatry, International Psychogeriatrics, and Psychiatry Research. The journals primarily focus on topics related to elderly individuals, covering areas such as affective disorders, geriatric psychiatry, mental health and social support, diagnosis and treatment of neuropsychiatric conditions, and epidemiology and intervention in psychiatry. This thematic emphasis aligns with the findings presented in the dual-map analysis shown in **Figure 6B**, reinforcing the interconnectedness of these research areas.

Keyword analysis is an essential methodology in the investigation of suicide among the elderly, offering profound insights into current research trends and focal areas. By examining frequently used keywords, researchers can identify key themes and pressing issues of concern. The keywords identified in this study—depression, suicidal ideation, suicide, older-adults, risk, risk-factors, prevalence, older adults, ideation, behavior, health, mental-health, life, age, people, prevention, symptoms, scale, population, elder—encompass crucial aspects such as the identification, treatment, and prevention of depression and suicide within the elderly population (**Figure 7A**). These keywords highlight the complex and comprehensive nature of research at the intersection of aging and suicide.

Suicide is categorized into suicidal ideation, suicide attempts, and suicidal behavior (56). The definition of suicidal ideation is still debated, generally encompassing negative thoughts about death or ending one’s own life (57). Passive suicidal ideation involves thoughts like wishing for death without specific plans, while active suicidal ideation includes intentions to end one’s life (58). A suicide attempt is defined as a self-destructive act with some intent to end one’s life (59). Suicidal behavior refers to suicidal deaths and includes various types of suicide attempts (60). With the global aging population on the rise, elderly suicide has become a focal point of our attention.

The main risk factors for suicide in the elderly include mental illnesses, physical illnesses, substance abuse, low social support, feelings of loneliness, marital status, financial pressure, previous suicide attempts, and having a first-degree relative with a history of mental illness or suicide (61). A study analyzing 28 risk factors for elderly suicide found that the most significant risk factors for attempts are depressive disorders, self-harm methods, psychotropic drug use, psychological factors, and disability, while completed suicides are primarily associated with mental disorders (depression, anxiety, bipolar disorder), physical illnesses, more stressful events, living alone, and being male (62). It should be noted that self-harm in the elderly is also a risk factor for suicide, with poisoning being the most common method of self-harm (63). Suicidal ideation in the elderly is associated with low educational levels, low quality of life, and high prevalence of mental illnesses (64). The risk factors for suicidal ideation differ by gender. For men, the main factors are limitations in daily living activities and chronic illnesses, while for women, increased suicidal ideation is primarily related to low economic satisfaction, more chronic illnesses, and poorer health conditions (65). A meta-analysis found that increased suicidal intent and planning distinguish high-lethality from low-lethality attempts, with high-lethality attempters being less impulsive, more likely to follow a plan, and more likely to be white (66). Another study indicated that being female, younger, and more impulsive is related to low-lethality attempts, whereas the opposite characteristics are associated with high-lethality attempts (67). Since elderly individuals often do not readily report suicidal ideation, the actual scope of suicide is frequently underestimated (68). Therefore, it is essential to focus on high-risk factors for suicide and evaluate them comprehensively rather than individually (58). In recent years, the “5D” factors—mental illness, disability, lack of social support, and highly lethal means—have increasingly become a focal point in the risk assessment of elderly suicide (69).

Elderly individuals with depression or depressive symptoms are significantly more likely to consider or attempt suicide (70). Additionally, meta-analyses have found that depression is associated with suicidal ideation, attempts, and deaths (71). Generally, in the absence of other influencing factors, the higher the degree of depression, the higher the level of suicidal ideation (72). Studies indicate that only 25% of psychiatric patients disclose their suicidal thoughts (73), and the rate of undiagnosed depression in the elderly is even higher. The Diagnostic and Statistical Manual of Mental Disorders, 5th Edition (DSM-5) outlines the criteria for diagnosing major depressive episodes, including core and secondary symptoms, duration, and severity; however, elderly individuals often focus on physical symptoms and may deny or overlook depressive feelings, making accurate identification of depression in older adults crucial (74). Another study demonstrated that social support mediates the relationship between depression and suicidal ideation (75). Furthermore, depression negatively impacts life satisfaction, increasing the risk of suicidal behavior, while social support can reduce suicidal behavior (76). The suicide risk due to cognitive, emotional, and physical symptoms in the elderly is lower than in younger individuals, but the relationship between depressive symptoms and suicide risk is adjusted by age in both groups (77). Both physical illnesses and physical symptoms increase the risk of suicide in the elderly, such as cancer, neurological diseases, chronic obstructive pulmonary disease (COPD), liver disease, joint disease, and pain, which are closely related to suicide (42). Another systematic review also has shown that somatic symptom disorders increase the risk of suicidal ideation and attempts (78). Additionally, both short-term and long-term sensory loss can increase the risk of suicidal ideation in the elderly (79). Elderly individuals with dementia or cognitive impairment have higher rates of depression and suicidal ideation (80, 81). A study in South Korea showed that both single and multiple types of elder abuse experiences are associated with an increased risk of late-life suicidal ideation (82).

Suicide is one of the mental health issues that carries significant stigma. Research has shown that stigmatizing attitudes toward individuals who have attempted suicide are widespread in many countries, particularly in low- and middle-income countries such as Irank (83, 84). Such stigmatization can profoundly influence individuals’ attitudes, beliefs, and behavioral patterns. Key characteristics of stigma associated with mental illness and suicide include fear, shame, negative beliefs about mental illness, social prejudice, social isolation, and discrimination. These factors pose significant barriers for individuals at risk of suicide to seek help (85). Seeking mental health support is crucial for preventing the deterioration of psychological issues and reducing the risk of suicide. However, a study reported that only 62% of individuals who attempted suicide sought mental health services in the year preceding their attempt (86). Evidence also indicates that reducing the stigma surrounding mental health issues is significantly associated with improved mental health outcomes, including a reduction in suicide rates (87). Therefore, increasing public awareness about suicide is a critical first step in addressing this public health challenge (88). For example, governments could enact anti-discrimination laws to reduce the stigma surrounding suicide and encourage individuals to seek help. Additionally, enhancing the understanding of suicide among the public and policymakers is equally vital (89, 90).

Between 2020 and 2050, the global population aged 80 and above is projected to reach 426 million, reflecting a rapidly aging world. Promoting and safeguarding the mental health of older adults is essential for healthy aging, with reducing suicide rates among the elderly being a critical priority. Public health initiatives must play a central role in monitoring and preventing suicide in this population. For policymakers and professionals in relevant fields, it is vital to identify specific risk factors and protective factors associated with suicide in older adults to develop targeted prevention strategies and action plans. Research identifies protective factors against suicide in the elderly as good physical and cognitive health, high quality of life, the ability to perform daily activities, marital status, strong social support, and religious faith, highlighting the need for comprehensive prevention strategies that address both mental health and social aspects (61). The reduction of functional impairment after treatment for depression is related to the reduction of suicidal ideation, particularly concerning thoughts of worthlessness (91). Firstly, it is essential to improve the recognition of depression in the elderly among professionals, especially in primary care. Secondly, combined treatment with antidepressant medication and cognitive-behavioral therapy (CBT) appears to be the most effective for depression (74). Outside of the hospital, performing arts can enrich elderly lives and foster new relationships, while media and community organizations can offer psychiatric education and support platforms for older adults (92). Research indicates that understanding mental health policies, raising public awareness and education, improving access to healthcare and screening tools, responsible media reporting, and restricting access to lethal means can all reduce the risk of suicide in the elderly (93). Home-based teletherapy has become increasingly popular and is particularly beneficial for elderly patients with mobility limitations (94). Suicide is the result of a complex interplay of multiple factors. Effective suicide prevention strategies and plans require active participation, coordination, and collaboration of all levels of society.

Our study has several limitations that should be acknowledged. First, in our study, we have used only the WOS database, which includes the vast majority of high-quality articles. However, we cannot exclude the possibility that a small number of articles may not have been included. In future research, we plan to incorporate multiple databases for analysis to minimize potential biases associated with the selection of a single database. Second, we recognize that non-English articles, particularly those not translated into English, may not be fully represented in the Web of Science database. This could introduce language bias and limit the generalizability of our findings, especially for research conducted in non-English-speaking regions. Third, the analysis relies on institutional affiliations provided by the authors, which are typically those listed in their publications. This reliance may restrict the representation of research conducted at individual institutions, particularly when an author’s affiliation is not listed or is incomplete. Lastly, our dataset ends in mid-2024, which may affect the citation counts for more recent publications. This limitation could result in the underrepresentation of the citation trends for newly published studies, potentially impacting the accuracy of the analysis, especially in examining emerging trends or overlooked topics. We suggest that these trends be revisited once updated data becomes available.

## 5 Conclusions

This bibliometric analysis highlights the leading role of the USA in suicide research among the elderly, with American institutions and scholars prominently shaping the field. Key findings stress the critical importance of addressing depression as a major risk factor for suicidal behavior, advocating for enhanced recognition, combined pharmacological and psychotherapeutic treatments, and robust social support systems. Additionally, effective prevention strategies should include improved mental health policies, public awareness, and access to healthcare, with home-based teletherapy offering a promising solution for those with mobility issues. A comprehensive approach that integrates these elements is essential for reducing elderly suicide rates and advancing intervention strategies.

## Data Availability

The raw data supporting the conclusions of this article will be made available by the authors, without undue reservation.
